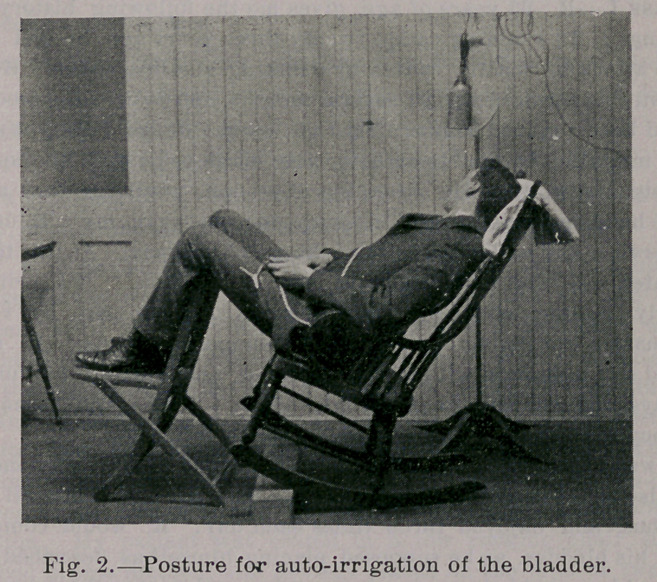# Bladder Gymnastics and Auto-Irrigation

**Published:** 1894-07

**Authors:** Byron H. Daggett

**Affiliations:** 258 Franklin Street; Buffalo, N. Y.


### BLADDER GYMNASTICS AND AUTO-IRRIGATION.

By BYRON H. DAGGETT, M. D., Buffalo, N. Y.

At the regular meeting of the Lake Erie Medical Association, July 15, 1892, I read an article describing a method of irrigation of the deep urethra and bladder without the use of a catheter, which was published in the Buffalo Medical and Surgical Journal, March, 1893. After the lapse of nearly two years' time, and the additional experience of continual tests, I feel warranted in asserting that nineteen out of twenty patients may be taught to irrigate in this way. The failures will be due to a very small stricture or a very large prostate.

I will briefly refer to a few notes taken since the publication of that paper, and preface these memoranda by repeating the technique of bladder irrigation without catheterization. The materials are a four-quart bag, a tube six feet long with a shut-off within easy reach. The tube is attached to the inlet of the double canula (Fig. 1), its bore being twenty per cent. larger than that of the outlet. The

Fig. 1.—The Daggett canula.

nozzle of the canula is introduced from one to two inches, according to the size of the meatus, and is made wedge-shaped, in order to fill the varying calibers of urethral meati. It is sufficiently long to be conveniently held in place by grasping the penis behind the glans, at the same time drawing the pendulous portion in line with the fixed urethra. The bag is filled with water, at a temperature of 115 degrees, to insure more than blood warmth as it flows, and is made bland by the addition of a little glycerine, mucilage, a few grains of salt or soda carb., and elevated two or three feet above the plane of the pelvis.

The patient must assume a reclining position—a reversed squatting posture—since flexure and gravity are essential factors. He may do this in an ordinary bath tub by resting his back along the incline at its head, so that the trunk is at an angle of forty-five degrees from the horizontal line, flexing the thighs at right angles with the body and supporting the legs at right angles with the thighs. If there is no bath tub at hand, a hip bath may be arranged for this purpose, or the patient may posture himself in a low rocking chair, tilted and blocked (Fig. 2), so that his body assumes the position described, his legs resting upon another chair or upon a stand.

Fig. 2.—Posture for auto-irrigation of the bladder.

The nozzle of the irrigator is then introduced, the penis grasped and drawn in line with the fixed urethra, the stop opened and the water allowed to run if necessary until the bag is empty ; if it has not passed into the bladder, try again. A peculiar feeling gives warning of the passing of the water through the posterior urethra, the return flow diminishes and escapes in a pulsating stream, when a finger of the right hand is placed over the exit, to divert the entire flow into the bladder, which at first resents the intrusion and ejects after receiving two to three ounces. Repeat this and the bladder becomes more tolerant each time. Three to four flushings are sufficient at each séance and the séances may be repeated three times daily if necessary. The novelty and comfort afforded by irrigation sometimes induce patients to overdo, at the beginning, before tolerance is established. The diminished, pulsating outflow would seem to indicate an anti- or retro-peristaltic action of the accelerator muscle. This process is a coaxing one, in which the gentle pressure of the continuous flow of the hot, non-irritating current and the posture described are essential conditions. The patient acquires a knack at the first success, which he realizes and which I can scarcely describe, that gives him an abiding faith in his ability to flush his bladder at will.

### MEMORANDA.

Case I.—R., 63 years of age, gives me the following history of his sufferings: In a mining camp, twenty-three years ago, he had a very severe attack of cystitis, caused by drinking alkaline water. He came home on this account, was ill several months, and never fully recovered. He had been confined to his room four weeks; drugs failing to bring relief, irrigation by double catheter was employed; still his condition grew steadily worse. His attending physician, realizing that a crisis was at hand, proposed to call in a surgeon to do cystotomy. R. declined this service, and called me to take charge of his case. At this time, he presented all the phenomena of septic infection. His urine was strongly alkaline, offensive, depositing one quarter part, by volume, solid matter, consisting of pus and inflammatory débris. R. readily learned self-irrigation without the catheter, and cleared his urine in five days, and was able to attend his office. There still remained a tendency to relapse, which was controlled by irrigation.

It was noted that irrigations not only warded off this tendency, but they also relieved soreness and pain, as he expressed himself, were luxurious, and enjoyed three times each day. R. would completely empty his bladder, as he supposed, placing himself as described. The first washing would show straw or amber color, the third would be clear. He passed into his bladder half a pint of hot milk, and maintained that, as hot milk was good for sore eyes, therefore it ought to be good for sore bladders. After micturating, he irrigated, and the first washing was very milky, the third showed up clear. These tests indicated residuum, which, becoming disturbed, caused cystitis. The prostate is not perceptibly enlarged, and he has never had retention. He firmly refused permission to pass the catheter to test the question of residual urine, alleging that he had already suffered sufficiently from the use of that instrument for irrigation.

After doing bladder gymnastics by three daily irrigations for six weeks, it is evident that this viscus is completely evacuated by normal urination, and, more than this, the rising stream is ejected with sufficient force to menace facial autonomy. Gymnastics of the lower urinary apparatus had relieved urinary stasis and its ever-attending threat.

Case II.—B., 20 years old, a railroad fireman, had been confined to his bed five days, suffering from prostatocystitis, caused by gonorrhea, passing thick decomposing urine every ten to fifteen minutes. He succeeded, in the second trial, in flushing the bladder. The swelling of a prostatic abscess interrupted or blocked deep irrigation for eighteen hours. Immediately following the rupture of the abscess, irrigation was successful and uninterrupted to the end. Pain practically ceased in three days, and within a week he could hold his water for six hours. For three days following the rupture, he passed masses of macerated blood coagula, which was made possible by flooding the bladder and evacuating its contents by normal urination. Recovery was speedy and complete.

Two cases of recurring attacks of orchitis due to chronic irritable posterior urethritis have been promptly relieved and relapses forestalled by irrigation pending other measures.

Dr. T. S. Stuart, of Buffalo, kindly permits me to include in this paper a synopsis of a report of one of his cases of prostatic hypertrophy, which caused stillicidium and retention of urine:

W., a house painter, 61 years old, states that for five years his urine has passed away by dribbling, constantly necessitating the use of protectives. April 20, 1894, he had a severe attack of strangury, and his physician being unable, after repeated efforts, to pass a catheter, palliated the symptoms by the administration of drugs. W. continued to suffer from distension and tenesmus. Dr. Stuart was called April 28th, and, after making diligent efforts, failed to pass a catheter. The case becoming urgent, Dr. Stuart says: "I determined to test the method proposed by Dr. Daggett—namely, relaxation by posture and hot water irrigation. My patient was seated in a low rocking chair, tilted as described in this method, and his legs placed on the tops of two other chairs. The canula was introduced, and within a short time he expressed a desire to urinate; the canula was then removed, and he passed nearly a pint of fluid. Irrigation was used twice daily for a week, then once daily since that time. Convalescence has been uninterupted, and his power to void urine has steadily improved. At present W. has no difficulty in urinating, dribbling has ceased, and he feels better than for several years past."

Another interesting phenomenon is developed by this process, and that is the restoration of impaired procreative functions.

After several years experience and two score cases as they occur in the rounds of a general practice, I feel warranted in asserting that more than ninety per cent. of these patients may be taught to irrigate the bladder without a catheter.

Success is attained by technique, posture and perseverance.

258 FRANKLIN STREET.

## Figures and Tables

**Fig. 1. f1:**
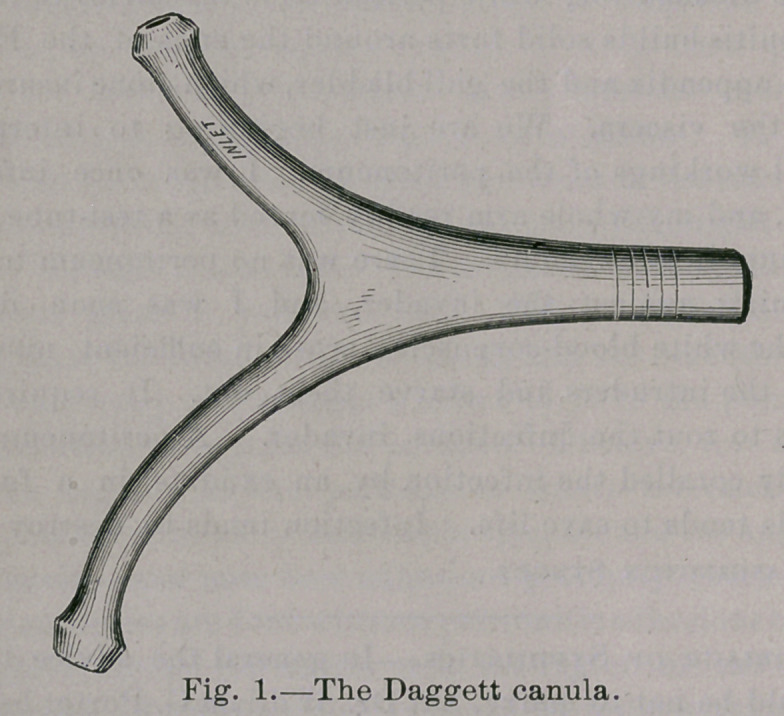


**Fig. 2. f2:**